# Synthesis of pyrimido[4,5-*b*]quinolindiones and formylation: ultrasonically assisted reactions

**DOI:** 10.1098/rsos.231128

**Published:** 2024-03-06

**Authors:** Jorge Trilleras, Andrés Charris-Molina, Alfredo Pérez-Gamboa, Paola Acosta-Guzman, Jairo Quiroga

**Affiliations:** ^1^ Programa de Química, Facultad de Ciencias Básicas, Universidad del Atlántico, Puerto Colombia 81007, Colombia; ^2^ Facultad de Ciencias Exactas y Naturales, Departamento de Química Inorgánica Analítica y Química Física, Universidad de Buenos Aires, Buenos Aires C1428EGA, Argentina; ^3^ Facultad de Ciencias, Departamento de Química, Universidad Nacional, Bogotá 11001, Colombia; ^4^ Heterocyclic Compounds Research Group, Department of Chemistry, Universidad del Valle, Cali 760032, Colombia

**Keywords:** β-diketones, pyrimidoquinolines, multicomponent synthesis, Vilsmeier–Haack reaction

## Abstract

Ultrasound-assisted synthesis of pyrimido‌quinolindione derivatives via a multicomponent reaction and subsequent formylation with Vilsmeier–Haack reagent were performed. Compounds were prepared by a one-pot method from aminopyrimidinones, dimedone and aromatic aldehydes through a Mannich-type reaction sequence, and then functionalized under ultrasound irradiation and Vilsmeier–Haack conditions to give β-chlorovinylaldehyde products. Ultrasonically assisted reactions, experimental simplicity, good yields without using metallic catalysts and the control of hazardous material release are features of this simple procedure.

## 1. Introduction

**Figure 1.**
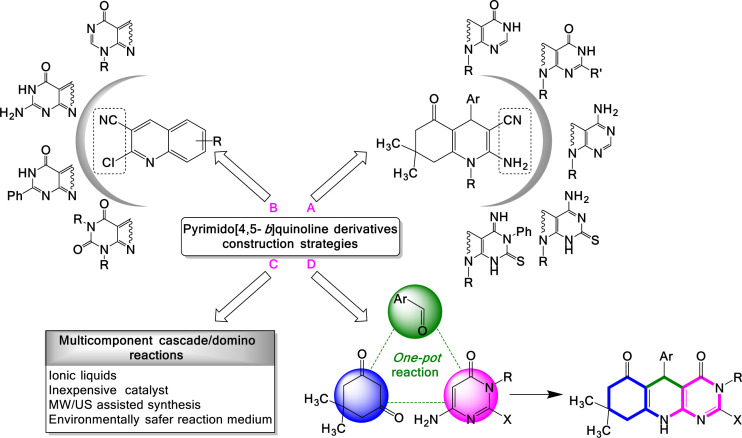
Convergent synthetic strategies towards the construction of pyrimido[4,5-*b*]quinoline derivatives.

Pyrimidoquinoline derivatives have been widely studied from several aspects, including synthetic procedures for obtaining detailed methodologies, reactions, precursors, catalysts, diversity, structural derivatization, analysis of biological activity and studies of potential applications as materials [1–19]. Convergent synthetic routes for the preparation of the pyrimido[4,5-*b*]quinoline template involved the construction of a pyrimidine ring on the pyridine core of the hydroquinoline ring. This is achieved through methods based on two-component reactions between appropriately C2/C3-functionalized hydroquinoline derivatives and amino or carbonitrile groups with carboxylic acids [1], oxalyl chloride [2], formic acid, acetic anhydride, formamide [3–5]; chloroacetyl chloride, guanidine hydrochloric salt, urea, thiourea [6,7]; and isothiocyanate [3,7] (figure 1A). Similarly, pyrimido[4,5-*b*]quinoline-4-ones, also known as deazaflavin analogues, have been prepared via amination and cyclization reactions of 2-chloroquinoline-3-carbonitriles with guanidine hydrochloride salts, urea and thiourea (figure 1B) [8–11].

Multicomponent reactions (MCRs) are well known for their advantages, including time reduction, atomic economy, operational simplicity and environmental friendliness. Combined with the wide commercial availability of reagents, particularly amines, aldehydes and ketones, these features have positioned MCRs as one of the most powerful techniques for the synthesis of polyfunctionalized heterocyclic systems. MCRs, in combination with microwave- or ultrasound-assisted synthesis, have contributed to the expansion of the molecular library of pyrimido[4,5-*b*]quinoline derivatives (figure 1C) [8–10,12–16], resulting in molecules with significant properties [1–7,12–16]. These studies have led to the development of greener and environmentally safer synthetic conditions [17–21] and reusable catalysts [22–25] (figure 1D). Tricyclic pyrimido[4,5-*b*]quinoline derivatives are often synthesized by two methods: one based on a two-component reaction using the quinoline or hydroquinoline nucleus (figure 1A and B) and the other by MCRs (figure 1C and D).

Our research group has developed synthetic strategies using small molecules via one-pot reactions to access highly functionalized heterocycles [26–30]. We efficiently used low-molecular-weight precursors and inexpensive, commercially available reagents to obtain pyrimido[4,5-*b*]quinoline [29,30]. Given the growing use of the cavitation phenomenon in chemical processes, particularly in organic transformations [31–33], we present the results of the synthesis of 5-aryl-tetrahydropyrimido[4,5-*b*]quinoline-4,6-dione derivatives in this study. This was achieved through a three-component, one-pot cyclocondensation, wherein the reaction was induced using both conventional (reflux, Method 1) and unconventional (ultrasound, Method 2) heating methods. 6-Amino-3-methyl-2-(methylthio)pyrimidin-4(3*H*)-one, 6-amino-2-methoxy-3-methylpyrimidin-4(3*H*)-one, dimedone and aromatic aldehydes were used to synthesize the target compounds.

## 2. Materials and methods

### 2.1. General aspects

Reagents and solvents were purchased from commercial sources. Ultrasonic irradiation was induced using an ultrasonic bath (Branson Model 1510; AC input 115 V, output 50 W, 1.9 l, mechanical timer, 60 min with continuous hold and heater switch, 47 KHz). The melting points of the purified compounds were measured using a scientific melting point apparatus (model IA 9100/Capillary). The uncorrected data have been reported previously. The experimental work was developed in the laboratory for the synthesis of heterocyclic compounds that are governed by the chemical safety standards in research laboratories under the guidelines of the integral waste management programme. The programme’s function is to provide adequate management of the waste produced in the daily processes and activities of the Universidad del Atlántico, thus complying with the current environmental regulations established by the country related to waste management. FT-IR spectra were recorded on a Shimadzu FTIR 8400 spectrophotometer (Scientific Instruments Inc., Seattle, WA, USA) using KBr disks with a resolution of 2 cm^−1^ and 16 scans (transmission mode 4000–500 cm^−1^). One- and two-dimensional NMR spectra were recorded on a Bruker Advance spectrophotometer using Tetramethylsilane (TMS) as the internal standard (δ, 0.0 ppm) and CDCl_3_ and DMSO-*d*_6_ as the solvents. NMR signals are in ppm, and coupling constants (*J*) are in Hz. ^1^H, ^13^C and Distortionless Enhancement by Polarizable Transfer (DEPT)-135 were recorded using a spectrophotometer at 400 and 100 MHz, respectively. Two-dimensional NMR spectra were obtained using heteronuclear single quantum correlation (HSQC) and heteronuclear multiple bond correlation (HMBC) experiments. Mass spectra were recorded on a Thermo Fisher Scientific GC-MS spectrometer (model DSQII) using a direct insertion probe and the electron impact ionization technique (70 eV). Microanalysis was performed using an elemental analyser (Agilent CHNS; Thermo Fisher Scientific Inc., Madison, WI, USA), and the values were within ±0.4% of the calculated values. The progress of all reactions was determined using thin-layer chromatography (TLC).

### 2.2. Synthesis of 5-aryl-tetrahydropyrimido[4,5-*b*]quinoline-4,6-dione derivatives **4** and **5**

6-Amino-3-methyl-2-(methylthio)pyrimidin-4(3*H*)-one, 6-amino-2-methoxy-3-methylpyrimidin-4(3*H*)-one (**1**), dimedone (**2**) and aldehyde aromatic (**3**) were combined in equimolar amounts (1.0 mmol) in acetic acid or ethyl alcohol (5.0 ml). The reaction mixture was then stirred at room temperature for 5 min. Heating was induced by reflux or ultrasound once the reaction mixture had been prepared. After the completion of the reaction (confirmed by TLC using dichloromethane (DCM)), it was cooled and filtered. The obtained solid was washed with cold ethyl alcohol (3.0 ml) and recrystallized from ethyl alcohol to give the 5-aryl-tetrahydropyrimido[4,5-*b*]quinoline-4,6-dione derivatives **4** and **5**.

### 2.3. Procedure for preparation of the β-chlorovinyl aldehyde derivatives **6** and **7**

The Vilsmeier–Haack (VH) reagent was prepared in two steps. POCl_3_ (0.11 mol, 9.4 ml) was initially cooled in an ice/water bath at 0°C under stirring in an argon atmosphere for 15 min. Then, *N*,*N*-dimethylformamide (DMF; 0.23 mol, 16.0 ml) was added dropwise while maintaining the temperature of the mixture at 0°C. After the complete addition of DMF, the mixture was stirred continuously for 30 min. Without altering the reactor conditions, 1.0 mmol of the corresponding pyrimido[4,5-*b*]quinoline derivative (**4** or **5** dissolved in DCM; 0.5 ml) was added for 10 min. The reaction mixture turned red and was irradiated in an ultrasonic bath for 2 h. The reaction was complete (confirmed by TLC using DCM), crushed ice was added and the mixture was stirred for another 15 min. After stirring and extraction with DCM (3 × 10 ml), the organic phase was dried over anhydrous MgSO_4_ and stirred for 15 min. The drying agent was filtered and the solvent was evaporated to yield the corresponding solid derivatives. Finally, the solids were purified using column chromatography on silica gel (hexane:ethyl acetate, 20:1).

#### 2.3.1. 6-Chloro-2-methoxy-8,8-dimethyl-4-oxo-5-phenyl-3,4,5,8,9,10-hexahydropyrimido[4,5-*b*]quinoline-7-carbaldehyde (**6a**)

Yellow solid; 70%. Mp: >300°C; IR (KBr, cm^−1^): 3503, 3130, 3026, 2960, 1727, 1656, 1562, 1450, 1407, 1099, 519. ^1^H NMR (400 MHz CDCl_3_) δ ppm: 1.13 (*s*, 3*H*, CH_3_), 1.48 (*s*, 3*H*, CH_3_), 2.35 (*d*, *J* = 17.6 Hz, 1*H*, CH_2_), 2.97 (*d*, *J* = 17.6 Hz, 1*H*, CH_2_), 3.31 (*s*, 3*H*, OCH_3_), 5.28 (*s*, 1*H*, CH), 7.24 (*t*, *J* = 7.2, 1*H*, CH), 7.31 (*t*, *J* = 7.4 Hz, 2*H*, CH), 7.36 (*d*, *J* = 7.2 Hz, 2*H*, CH), 9.66 (*s*, 1*H*, CHO), 11.78 (*s*, 1*H*, NH), 13.17 (*s*, 1*H*, NH); ^13^C NMR (100 MHz, CDCl_3_) δ ppm: 27.3 (CH_3_), 27.5 (CH_3_), 28.9 (CH), 33.0 (C), 37.5 (OCH_3_), 49.4 (CH_2_), 91.2 (C), 112.4 (C), 126.0 (C), 127.3 (CH), 127.7 (2× CH), 128.7 (2× CH), 141.2 (C), 142.2 (C), 145.8 (C), 147.8 (C), 151.2 (C), 162.2 (C), 192.0 (CHO). MS (70 eV) *m/z* (*I_r_*%): 398.2 (2, MH^+^). M.F.: C_21_H_20_ClN_3_O_3_, analysis calculated for C_21_H_20_ClN_3_O_3_ (397.12): C, 63.40%; H, 5.07%; N, 10.56%. Found: C, 63.61%; H, 5.12%; N, 10.69%.

#### 2.3.2. 6-Chloro-2-methoxy-5-(4-methoxyphenyl)-8,8-dimethyl-4-oxo-3,4,5,8,9,10-hexahydropyrimido[4,5-*b*]quinoline-7-carbaldehyde (**6b**)

Yellow solid; 60%. Mp: >300°C; IR (KBr, cm^−1^): 3509, 3062, 2959, 2860, 1702, 1646, 1561, 1464, 1419, 1078, 580. ^1^H NMR (400 MHz CDCl_3_) δ ppm: 1.01 (*s*, 3*H*, CH_3_), 1.05 (*s*, 3*H*, CH_3_), 2.32 (*dd*, *J* = 17.6 Hz, 2*H*, CH_2_), 2.89 (*s*, 3*H*, OCH_3_), 3.21 (*s*, 3*H*, OCH_3_), 4.26 (*s*, 1*H*, CH), 6.81 (*t*, *J* = 7.4 Hz, 2*H*, CH), 7.04 (*d*, *J* = 7.2 Hz, 2*H*, CH), 9.48 (*s*, 1*H*, CHO), 10.08 (*s*, 1*H*, NH), 12.23 (*s*, 1*H*, NH). ^13^C NMR (100 MHz, CDCl_3_) δ ppm: 27.3 (CH_3_), 27.6 (CH_3_), 28.9 (CH), 33.0 (C), 36.7 (OCH_3_), 49.4 (CH_2_), 55.3 (OCH_3_), 91.5 (C), 112.4 (C), 114.1 (2× CH), 126.2 (C), 128.8 (2× CH), 134.4 (C), 141.1 (C), 145.6 (C), 147.7 (C), 151.2 (C), 158.7 (C), 162.3 (C), 192.0 (CHO). MS (70 eV) *m/z* (*I_r_*%): 429.2 (18), 428.2 (43, MH^+^), 427.2 (12), 412.2 (100). M.F.: C_22_H_22_ClN_3_O_4_, analysis calculated for C_22_H_22_ClN_3_O_4_ (427.13): C, 61.76%; H, 5.18%; N, 9.82%. Found: C, 61.86%; H, 5.02%; N, 9.97%.

#### 2.3.3. 6-Chloro-2-methoxy-8,8-dimethyl-4-oxo-5-(3,4,5-trimethoxyphenyl)-3,4,5,8,9,10-hexahydropyrimido[4,5-*b*]quinoline-7-carbaldehyde (**6c**)

Yellow solid; 79%. Mp = 287–290°C; IR (KBr, cm^−1^): 3509, 2959, 2860, 1702, 1646, 1609, 1561, 1510, 1031, 519. ^1^H NMR (400 MHz CDCl_3_) δ ppm: 1.16 (*s*, 3*H*, CH_3_), 1.46 (*s*, 3*H*, CH_3_), 2.35 (*d*, *J* = 17.7 Hz, 1*H*, CH_2_), 2.93 (*d*, *J* = 17.6 Hz, 1*H*, CH2), 3.30 (*s*, 3*H*, OCH_3_), 3.80 (*s*, 9*H*, OCH_3_), 5.21 (*s*, 1*H*, CH), 6.56 (*s*, 2*H*, CH), 9.63 (*s*, 1*H*, CHO), 11.74 (*s*, 1*H*, NH), 13.11 (*s*, 1*H*, NH). ^13^C NMR (100 MHz, CDCl_3_) δ ppm: 27.4 (CH_3_), 27.5 (CH_3_), 28.9 (CH), 33.0 (C), 37.5 (OCH_3_), 49.4 (CH_2_), 56.4 (2× OCH_3_), 60.9 (OCH_3_), 91.1 (C), 105.2 (2× CH), 112.4 (C), 125.9 (C), 137.5 (C), 137.7 (C), 141.2 (C), 146.0 (C), 147.9 (C), 151.2 (C), 153.3 (C), 162.3 (C), 192.0 (CHO). MS (70 eV) *m/z* (*I_r_*%): 488 (1, MH^+^), (75.7), 457.2 (44), 57.1 (100). M.F.: C_24_H_26_ClN_3_O_6_, analysis calculated for C_24_H_26_ClN_3_O_6_ (487.15): C, 59.08%; H, 5.37%; N, 8.61%. Found: C, 59.27%; H, 5.17%; N, 8.77%.

#### 2.3.4. 6-Chloro-5-(4-chlorophenyl)-2-methoxy-8,8-dimethyl-4-oxo-3,4,5,8,9,10-hexahydropyrimido[4,5-*b*]quinoline-7-carbaldehyde (**6d**)

Yellow solid; 71%. Mp: >300°C; IR (KBr, cm^−1^): 3529, 1722, 1660, 1521, 1108, 529. ^1^H NMR (400 MHz CDCl_3_) δ ppm: 1.13 (*s*, 3*H*, CH_3_), 1.48 (*s*, 3*H*, CH_3_), 2.36 (*d*, *J* = 17.2 Hz, 1*H*, CH_2_), 2.97 (*d*, *J* = 17.4 Hz, 1*H*, CH_2_), 3.30 (*s*, 3*H*, OCH_3_), 5.23 (*s*, 1*H*, CH), 7.27–7.31 (*m*, 4*H*, CH), 9.66 (*s*, 1*H*, CHO), 11.74 (*s*, 1*H*, NH), 13.14 (*s*, 1*H*, NH). ^13^C NMR (100 MHz, CDCl_3_) δ ppm: 27.3 (CH_3_), 27.5 (CH_3_), 29.0 (CH), 33.0 (C), 37.0 (OCH_3_), 49.3 (CH_2_), 90.7 (C), 112.6 (C), 125.5 (C), 128.9 (2× CH), 129.2 (2× CH), 133.1 (C), 140.7 (C), 141.3 (C), 146.2 (C), 147.3 (C), 151.1 (C), 162.2 (C), 192.2 (CHO). MS (70 eV) *m/z* (*I_r_*%): 432 (60, MH^+^), 431 (16), 433 (40), 416 (72), 320 (96), 284 (100). M.F.: C_21_H_19_Cl_2_N_3_O_3_, analysis calculated for C_21_H_19_Cl_2_N_3_O_3_ (431.08): C, 58.35%; H, 4.43%; N, 9.72%. Found: C, 58.55%; H, 4.21%; N, 9.86%.

#### 2.3.5. 6-Chloro-3,8,8-trimethyl-2-(methylthio)-4-oxo-5-phenyl-3,4,5,8,9,10-hexahydropyrimido[4,5-*b*]quinoline-7-carbaldehyde (**7a**)

Yellow solid; 70%. Mp = 229–232°C; IR (KBr, cm^−1^): 3455, 3058, 2958, 2933, 2872, 1661, 1638, 1524, 1454, 1411, 1063, 508. ^1^H NMR (400 MHz CDCl_3_) δ ppm: 1.12 (*s*, 3*H*, CH_3_), 1.43 (*s*, 3*H*, CH_3_), 2.29 (*d*, *J* = 17.4 Hz, 1*H*, CH_2_), 2.60 (*s*, 3*H*, SCH_3_), 2.94 (*d*, *J* = 17.4 Hz, 1*H*, CH_2_), 3.43 (*s*, 3*H*, NCH_3_), 5.31 (*s*, 1*H*, CH), 7.21 (*t*, *J* = 7.3 Hz, 1*H*, CH), 7.28 (*d*, *J* = 6.1 Hz, 2*H*, CH), 7.39 (*d*, *J* = 8.6 Hz, 2*H*, CH), 9.64 (*s*, 1*H*, CHO), 12.43 (*s*, 1*H*, NH). ^13^C NMR (100 MHz, CDCl_3_) δ ppm: 15.0 (SCH_3_), 27.7 (CH_3_), 29.0 (CH_3_), 30.2 (NCH_3_), 33.0 (C), 38.3 (CH), 49.4 (CH_2_), 98.0 (C), 111.1 (C), 126.0 (C), 127.0 (CH), 127.8 (2× CH), 128.5 (2× CH), 136.4 (C), 142.2 (C), 145.5 (C), 149.7 (C), 160.7 (C), 162.2 (C), 190.9 (CHO). MS (70 eV) *m/z* (*I_r_*%): 428.0 (43, MH^+^), 421 (100), 350 (23), 306 (54), 88 (56). M.F.: C_22_H_22_ClN_3_O_2_S, analysis calculated for C_22_H_22_ClN_3_O_2_S (427.11): C, 61.75%; H, 5.18%; N, 9.82%. Found: C, 61.88%; H, 5.10%; N, 9.89%.

#### 2.3.6. 6-Chloro-5-(4-methoxyphenyl)-3,8,8-trimethyl-2-(methylthio)-4-oxo-3,4,5,8,9,10-hexahydropyrimido[4,5-*b*]quinoline-7-carbaldehyde (**7b**)

Yellow solid; 70%. Mp = 220–222°C; IR (KBr, cm^−1^): 3475, 3014, 2954, 2835, 1668, 1637, 1530, 1456, 1425, 1080, 508. ^1^H NMR (400 MHz CDCl_3_) δ ppm: 1.12 (*s*, 3*H*, CH_3_), 1.43 (*s*, 3*H*, CH_3_), 2.28 (*d*, *J* = 17.4 Hz, 1*H*, CH_2_), 2.60 (*s*, 3*H*, SCH_3_), 2.93 (*d*, *J* = 17.3 Hz, 1*H*, CH_2_), 3.43 (*s*, 3*H*, NCH_3_), 3.77 (*s*, 3*H*, OCH_3_), 5.25 (*s*, 1*H*, CH), 6.81 (*d*, *J* = 8.7 Hz, 2*H*, CH), 7.29 (*d*, *J* = 6.5 Hz, 2*H*, CH), 9.64 (*s*, 1*H*, CHO), 12.43 (*s*, 1*H*, NH). ^13^C NMR (100 MHz, CDCl_3_) δ ppm: 15.0 (SCH_3_), 27.7 (CH_3_), 29.0 (CH_3_), 30.2 (NCH_3_), 33.0 (C), 37.4 (CH), 49.3 (CH_2_), 55.2 (OCH_3_), 98.3 (C), 111.0 (C), 114.0 (2× CH), 126.2 (C), 128.8 (2× CH), 134.6 (C), 142.0 (C), 145.5 (C), 149.6 (C), 158.4 (C), 160.7 (C), 162.4 (C), 190.9 (CHO). MS (70 eV) *m/z* (*I_r_*%): 458 (44, MH^+^), 442 (75), 306 (57), 97 (63), 83 (69), 57 (100), 55 (79), 43 (82). M.F.: C_23_H_24_ClN_3_O_3_S, analysis calculated for C_23_H_24_ClN_3_O_3_S (457.12): C, 60.32%; H, 5.28%; N, 9.18%. Found: C, 60.48%; H, 5.00%; N, 9.08%.

#### 2.3.7. 6-Chloro-3,8,8-trimethyl-2-(methylthio)-4-oxo-5-(3,4,5-trimethoxyphenyl)-3,4,5,8,9,10-hexahydropyrimido[4,5-*b*]quinoline-7-carbaldehyde (**7c**)

Yellow solid; 74%. Mp = 236–239°C; IR (KBr, cm^−1^): 3464, 2996, 2883, 2835, 1665, 1635, 1525, 1456, 1417, 1008, 479. ^1^H NMR (400 MHz CDCl_3_) δ ppm: 1.16 (*s*, 3*H*, CH_3_), 1.44 (*s*, 3*H*, CH_3_), 2.31 (*d*, *J* = 17.4 Hz, 1*H*, CH_2_), 2.61 (*s*, 3*H*, SCH_3_), 2.94 (*d*, *J* = 17.3 Hz, 1*H*, CH_2_), 3.46 (*s*, 3*H*, NCH_3_), 3.81 (*s*, 6*H*, OCH_3_), 5.26 (*s*, 1*H*, CH), 6.60 (*s*, 3*H*, OCH_3_), 7.29 (*s*, 2*H*, CH), 9.65 (*s*, 1*H*, CHO), 12.40 (*s*, 1*H*, NH). ^13^C NMR (100 MHz, CDCl_3_) δ ppm: 15.0 (SCH_3_), 27.6 (CH_3_), 29.0 (CH_3_), 30.3 (NCH_3_), 33.0 (C), 38.3 (CH), 49.4 (CH_2_), 56.1 (2× OCH_3_), 60.8 (OCH_3_), 97.8 (C), 104.9 (2× CH), 111.1 (C), 125.9 (C), 137.0 (C), 137.9 (C), 142.1 (C), 145.4 (C), 149.7 (C), 153.1 (C), 160.7 (C), 162.6 (C), 191.0 (CHO). MS (70 eV) *m/z* (*I_r_*%): 518 (6, MH^+^), 466 (9), 236 (19), 111 (37), 97 (68), 86 (73), 57 (100), 43 (92). M.F.: C_25_H_28_ClN_3_O_5_S, analysis calculated for C_25_H_28_ClN_3_O_5_S (517.14): C, 57.97%; H, 5.45%; N, 8.11%. Found: C, 57.74%; H, 5.19%; N, 8.51%.

#### 2.3.8. 6-Chloro-5-(4-chlorophenyl)-3,8,8-trimethyl-2-(methylthio)-4-oxo-3,4,5,8,9,10-hexahydropyrimido[4,5-*b*]quinoline-7-carbaldehyde (**7d**)

Yellow solid; 71%. Mp = 218–220°C; IR (KBr, cm^−1^): 3478, 3128, 2872, 1727, 1562, 1450, 1100, 519. ^1^H NMR (400 MHz CDCl_3_) δ ppm: 1.11 (*s*, 3*H*, CH_3_), 1.43 (*s*, 3*H*, CH_3_), 2.29 (*d*, *J* = 17.4 Hz, 1*H*, CH_2_), 2.60 (*s*, 3*H*, SCH_3_), 2.94 (*d*, *J* = 18.8 Hz, 1*H*, CH_2_), 3.43 (*s*, 3*H*, NCH_3_), 5.25 (*s*, 1*H*, CH), 7.24 (*d*, *J* = 8.6 Hz, 2*H*, CH), 7.30 (*d*, *J* = 8.4 Hz, 2*H*, CH), 9.66 (*s*, 1*H*, CHO), 12.41 (*s*, 1*H*, NH). ^13^C NMR (100 MHz, CDCl_3_) δ ppm: 15.0 (SCH_3_), 27.6 (CH_3_), 29.0 (CH_3_), 30.2 (NCH_3_), 33.0 (C), 37.7 (CH), 49.3 (CH_2_), 97.4 (C), 111.2 (C), 125.6 (C), 128.7 (2× CH), 129.2 (2× CH), 132.7 (C), 140.8 (C), 142.4 (C), 144.9 (C), 149.8 (C), 160.6 (C=O), 162.8 (C), 191.2 (CHO). MS (70 eV) *m/z* (*I_r_*%): 462 (6, MH^+^), 448 (32), 446 (43), 306 (33), 97 (63), 83 (64), 57 (100), 43 (89). M.F.: C_22_H_21_Cl_2_N_3_O_2_S, analysis calculated for C_22_H_21_Cl_2_N_3_O_2_S (461.07): C, 57.15%; H, 4.58%; N, 9.09%. Found: C, 57.42%; H, 4.73%; N, 9.28%.

#### 2.3.9. 4,6-Dichloro-5-(4-chlorophenyl)-8,8-dimethyl-2-(methylthio)-5,8,9,10-tetrahydropyrimido[4,5-*b*]quinoline-7-carbaldehyde (**9**)

The VH reagent was prepared by mixing DMF (0.3 mol, 23 ml) added dropwise to ice-cold phosphoryl chloride (0.2 mol, 18.7 ml) following the procedure detailed in §3.3, to yield **9** as a yellow solid (72% yield). Mp = 214–216°C; IR (KBr, cm^−1^): 3448, 2954, 2858, 1643, 1564, 1515, 1450, 1078, 520. ^1^H NMR (400 MHz CDCl_3_) δ ppm: 1.03 (*s*, 3*H*, CH_3_), 1.44 (*s*, 3*H*, CH_3_), 2.31 (*d*, *J* = 17.5 Hz, 1*H*, CH_2_), 2.55 (*s*, 3*H*, SCH_3_), 2.95 (*d*, *J* = 17.4 Hz, 1*H*, CH_2_), 5.37 (*s*, 1*H*, CH), 7.21 (*d*, *J* = 8.5 Hz, 2*H*, CH), 7.28 (*d*, *J* = 9.0 Hz, 2*H*, CH), 9.68 (*s*, 1*H*, CHO), 12.72 (*s*, 1*H*, NH). ^13^C (100 MHz, CDCl_3_) δ ppm: 14.4 (SCH_3_), 27.4 (CH_3_), 28.5 (CH_3_), 33.0 (C), 39.7 (CH), 49.0 (CH_2_), 109.0 (C), 112.5 (C), 124.6 (C), 129.0 (2× CH), 129.1 (2× CH), 133.5 (C), 138.5 (C), 141.8 (C), 143.4 (C), 155.5 (C), 158.7 (C), 171.8 (C), 191.1 (CHO). MS (70 eV) *m*/*z* (*I_r_*%): 466 (30, MH^+^), 456 (12), 454 (38), 452 (100). M.F.: C_21_H_18_Cl_3_N_3_OS, analysis calculated for C_21_H_18_Cl_3_N_3_OS (465.02): C, 54.03%; H, 3.89%; N, 9.00%. Found: C, 54.23%; H, 3.87%; N, 9.12%.

#### 2.3.10. 6-Chloro-5-(4-chlorophenyl)-7-(hydrazineylidenemethyl)-3,8,8-trimethyl-2-(methylthio)-5,8,9,10-tetrahydropyrimido[4,5-*b*]quinolin-4(3*H*)-one (**10**)

A mixture of 6-chloro-5-(4-chlorophenyl)-3,8,8-trimethyl-2-(methylthio)-4-oxo-3,4,5,8,9,10-hexahydropyrimido[4,5-*b*]quinoline-7-carbaldehyde (**7d**, 1 mmol) and hydrazine hydrate (1.0 mmol) was subjected to microwave irradiation (MWI) for 3 min using a focused microwave reactor (CEM Corporation, Matthews, NC, USA; 300 W and 120°C). The solid product was filtered and purified by crystallization from ethyl alcohol to obtain title compound **10** as an orange solid (76% yield). Mp >300°C; IR (KBr, cm^−1^): 3476, 3351, 3300, 2955, 2900, 1602, 1564, 1443, 1414, 1088, 600. ^1^H NMR (400 MHz DMSO-*d*_6_) δ ppm: 1.17 (*s*, 3*H*, CH_3_), 1.21 (*s*, 3*H*, CH_3_), 2.46 (*s*, 3*H*, SCH_3_), 2.62–2.50 (*m*, 2*H*, CH_2_), 3.30 (*s*, 3*H*, NCH_3_), 6.13 (*s*, 1*H*, CH), 6.51 (*s*, 2*H*, NH_2_), 6.94 (*d*, *J* = 8.59 Hz, 2*H*, CH), 7.20 (*d*, *J* = 8.39 Hz, 2*H*, CH), 7.39 (*s*, 1*H*, CH=N), 12.38 (*s*, 1*H*, NH). ^13^C NMR (100 MHz, DMSO-*d*_6_) δ ppm: 14.0 (SCH_3_), 28.1 (CH_3_), 28.3 (CH_3_), 29.9 (NCH_3_), 30.4 (C), 40.0 (CH), 49.5 (CH_2_), 91.5 (C), 99.6 (C), 123.0 (CH=N), 124.4 (C), 127.7 (C), 129.6 (2× CH), 130.0 (2× CH), 132.7 (C), 139.4 (C), 146.0 (C), 157.9 (C), 159.2 (C), 162.0 (C). MS (70 eV) *m*/*z* (*I_r_*%): 476 (60, MH^+^), 301 (100), 102 (80). M.F.: C_22_H_23_Cl_2_N_5_OS, analysis calculated for C_22_H_23_Cl_2_N_5_OS (475.10): C, 55.46%; H, 4.87%; N, 14.70%. Found: C, 55.36%; H, 4.73%; N, 14.52%.

#### 2.3.11. 7-(((2-Aminophenyl)imino)methyl)-6-chloro-5-(4-chlorophenyl)-3,8,8-trimethyl-2-(methylthio)-5,8,9,10-tetrahydropyrimido[4,5-*b*]quinolin-4(3*H*)-one (**11**)

A mixture of 6-chloro-5-(4-chlorophenyl)-3,8,8-trimethyl-2-(methylthio)-4-oxo-3,4,5,8,9,10-hexahydropyrimido[4,5-*b*]quinoline-7-carbaldehyde (**7d**, 1 mmol) and *o*-phenylenediamine (1.0 mmol) was subjected to MWI (300 W and 150°C) for 3 min. The solid product was filtered and purified by crystallization from ethyl alcohol to obtain **11** as a red solid (70% yield). Mp >300°C. IR (KBr, cm^−1^): 3664, 3400, 3342, 2928, 1662, 1636, 1598, 1516, 1415, 1083, 623. ^1^H NMR (400 MHz DMSO-*d*_6_) δ ppm: 1.01 (*s*, 3*H*, CH_3_), 1.34 (*s*, 3*H*, CH_3_), 2.27 (*d*, *J* = 17.4 Hz, 1*H*, CH_2_), 2.53 (*s*, 3*H*, SCH_3_), 2.93 (*d*, *J* = 17.3 Hz, 1*H*, CH_2_), 3.28 (*s*, 3*H*, NCH_3_), 5.04 (*s*, 1*H*, CH), 5.07 (*s*, 2*H*, NH_2_), 6.61 (*t*, *J* = 7.2 Hz, 1*H*, CH), 6.80 (*d*, *J* = 7.0 Hz, 1*H*, CH), 6.95 (*t*, *J* = 7.4 Hz, 1*H*, CH), 7.21 (*d*, 1*H*, CH), 7.24 (*d*, 2*H*, CH), 7.31 (*d*, *J* = 8.5 Hz, 2*H*, CH), 8.48 (*s*, 1*H*, CH=N), 13.72 (*s*, 1*H*, NH). ^13^C NMR (100 MHz, DMSO-*d*_6_) δ ppm: 14.7 (SCH_3_), 27.1 (CH_3_), 27.8 (CH_3_), 29.7 (NCH_3_), 33.4 (C), 37.4 (CH), 47.9 (CH2), 94.6 (C), 111.3 (C), 115.4 (CH), 116.3 (CH), 116.9 (CH), 125.4 (C), 126.8 (CH), 128.3 (2× CH), 129.1 (2× CH), 131.2 (C), 134.3 (C), 136.2 (C), 138.7 (C), 141.6 (C), 142.5 (C), 150.4 (C), 154.2 (C), 159.6 (C), 162.5 (C). MS (70 eV) *m*/*z* (*I_r_*%): 552.1 (12, MH^+^). M.F.: C_28_H_27_Cl_2_N_5_OS, analysis calculated for C_28_H_27_Cl_2_N_5_OS (551.13): C, 60.87%; H, 4.93%; N, 12.68%. Found: C, 60.90%; H, 4.80%; N, 12.58%.

## 3. Results and discussion

**Scheme 1.**
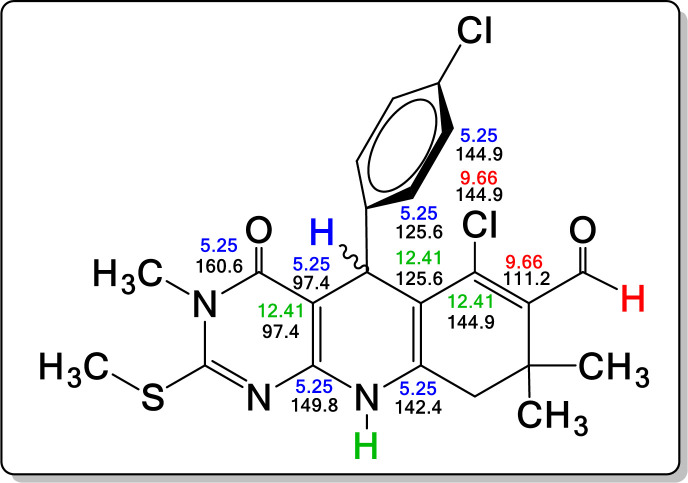
Synthesis of 5-aryl-5,8,9,10-tetrahydropyrimido[4,5-*b*]quinoline-4,6-dione derivatives **4** and **5**.

Various strategies have been reported for obtaining pyrimido[4,5-*b*]quinoline templates under thermal conditions (reflux or microwave irradiation), yielding improved results for a variety of amino-pyrimidines, aldehydes and active methylenes. These strategies allow the incorporation of different substitutes at positions 2, 3 and 5 of the key compounds, as outlined in figure 1. This expansion of the molecular library allows the evaluation of shifts in the chemical and biological properties of pyrimido[4,5-*b*]quinoline derivatives. Interest in these compounds for their potential applications, including as intermediaries for obtaining novel heterocyclic systems, has motivated organic chemists to explore synthetic procedures for the development of new pyrimido[4,5-*b*]quinoline-based structures. With this background from the literature and the experience of our research group in MCRs, this work began by exploring one-pot combinations with different substrates. Not all experiments using EWG- and ERG-substituted aromatic aldehydes yielded satisfactory results. In some cases, the yields were very low and not enough for structural characterization; in others, it was not possible to isolate and purify the product. According to the obtained yields, the influence of the substituents was not significant. The reduction in time is substantial when the reaction is assisted by ultrasound radiation; therefore, we consider that the influence on the reaction rate and increase in the yield of compounds **4** and **5** is due to the cavitation phenomenon. Here, we report the experimental results of a simple and fast procedure for the efficient synthesis of the 5-aryl-tetrahydropyrimido[4,5-*b*]quinoline-4,6-dione derivatives **4** and **5** (scheme 1). Equimolar amounts of 6-aminopyrimidinone (**1**), aromatic aldehyde (**2**) and dimedone (**3**) (cyclic β-diketone) were combined in a single step. To maintain environmentally friendly reaction conditions, we avoided the use of toxic solvents, heterogeneous catalysts or special conditions. The influence of the solvent and heating mechanism (reflux and ultrasound) on the yield and reaction time for obtaining derivatives **4** and **5** was evaluated. The results are presented in table 1.

**Table 1.** Reaction conditions for the synthesis of compounds **4** and **5** via scheme 1.

**entry**	**compound 4**						**compound 5**	
	**reflux**				**ultrasound**		**ultrasound**	
	**ethanol**		**acetic acid**		**acetic acid**		**acetic acid**	
	**time (h)**	**yield (%)**	**time (h)**	**yield (%)**	**time (min)**	**yield (%)**	**time (min)**	**yield (%)**
**a**	6	68	3	64	10	74	15	74
**b**	6	65	3	60	15	70	15	72
**c**	3	70	3	68	5	76	7	77
**d**	3	67	4	65	5	73	7	73

To implement the standard reaction conditions, the 5-aryl-tetrahydropyrimido[4,5-*b*]quinoline-4,6-dione derivative **4** was synthesized using both heating mechanisms (reflux and ultrasound). Polar protic solvents favour MCRs; therefore, the experiments were initiated by combining the reactants in ethyl alcohol and stirring at room temperature. No reactions were observed under these conditions. The reaction progressed when the temperature increased to 60°C. In both experiments, using ethanol or acetic acid as the solvent at 60°C, the product was isolated in a low yield (≤40%). Therefore, to improve the yield of the 5-aryl-tetrahydropyrimido[4,5-*b*]quinoline-4,6-dione derivative **4**, the reaction mixture was refluxed. It was also observed that using acetic acid as the solvent resulted in shorter reaction times, although the yields remained constant. These results show progress in optimizing the reaction conditions, increasing mass efficiency, reducing by-products and minimizing environmental impact.

**Scheme 2.**
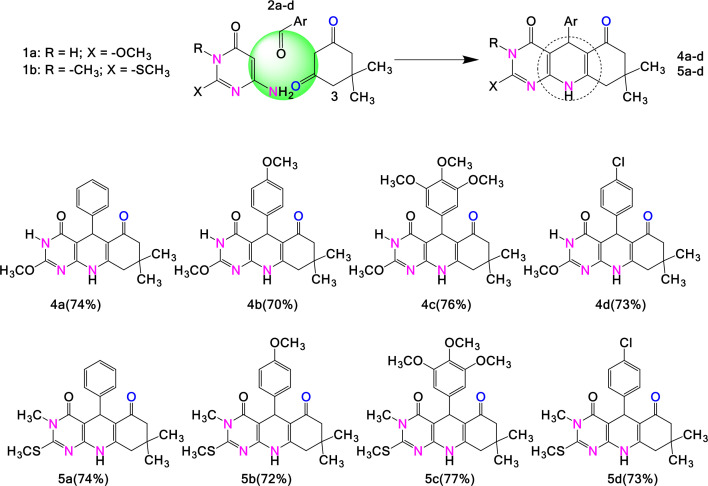
Possible mechanistic route for the formation of pyrimido[4,5-*b*]quinoline derivatives **4** and **5**.

The MCR process is complemented by cavitation to accelerate the reaction and build multicyclic compounds [32–34]. Therefore, the reaction was induced by ultrasound irradiation (USI), and the product was obtained in good yield within a few minutes. As a result, the standard reaction conditions were established as ultrasonically assisted synthesis using acetic acid as a solvent to generate 5-aryl-tetrahydropyrimido[4,5-*b*]quinoline-4,6-dione derivatives **4a–4d** and **5a–5d** with good performances (table 1). Purification of the product was simple and did not require column chromatography. Compounds **4** and **5** were synthesized following a Mannich-type reaction sequence. Scheme 2 shows the plausible mechanism for their formation.

The combination begins with an ultrasound-assisted Knoevenagel reaction between 5,5-dimethyl-1,3-cyclohexanedione (cyclic ketone nucleophilic) and aromatic aldehyde to provide adduct I. Then, adduct I undergoes cyclization through a Michael-type reaction with 6-aminopyrimidinone **1**, leading to the formation of the corresponding three-cyclic compounds **4** and **5**. These types of transformations have been previously studied and reported [16–25], and we have gained experience in this area [26,29,35]. Our results indicate that these MCRs follow a similar synthetic sequence, even with pyrazole nuclei [27,28]. Therefore, we decided to synthesize compounds **4** and **5** and isolate and purify them without characterization for further transformations. This work contributes to expanding the synthetic alternatives and molecular library of pyrimidoquinoline derivatives from a variety of aminopyrimidin-4-ones [16–25]. The synergy between acetic acid as a solvent, energy transfer agent and ultrasound radiation accelerated dehydration in the reaction. As shown in table 2, MCRs, closely related to this study, show better performance and atomic economy, and combine well with catalysts and solvents. Pyrimido[4,5-*b*]quinoline derivatives prepared under catalytic conditions had higher yields, in most cases requiring conventional heating. However, catalyst-free MCRs exhibit good performance and simple work-up. This work was carried out at a microscale, similar to that reported in the literature. MCRs for obtaining pyrimido[4,5-b]quinolinedione derivatives, including our work, have been reported in combinations of substrates in equimolar amounts of 1 mmol [9–30]. This is unlike some two-component synthetic techniques that scale to 0.01 mol and work well on a gram scale (approx. 5 g) [2–7].

**Table 2.** Performance comparisons of synthesis of pyrimido[4,5-*b*]quinoline derivatives in this work and in literature.

**technique**	**heating**	**catalyst**	**solvent**	**T (°C)**	**time (min)**	**yield (%)**	**ref.**
two-component	conventional	CuI, NaOH	DMSO	100	270–300	75–85	[8]
	MW	CuCl_2_, K_2_CO_3_	DMF	150	30	60–86	[9]
	conventional	CuCl_2_, K_2_CO_3_	DMF	—	240–300	62–78	[9]
	conventional	DMF, POCl_3_	DMF	60	15–270	82–98	[10]
multi-component	ultrasound (US)	—	acetic acid	—	5–15	70–77	this work
	US	Fe_3_O_4_ @SiO_2_-SnCl_4_	H_2_O	60	50–90	97–99	[16]
	MW	—	glycol	198	4–7	87–95	[17]
	conventional	C_6_H_5_CH_2_N(Cl)(C_2_H_5_)_3_	H_2_O	80–90	720–1200	62–73	[18]
	conventional	SBA-15-Pr-SO_3_H	H_2_O–EtOH	90	1–6	74–94	[19]
	US	—	EtOH	50	1.5–3	96–99	[20]
	US	piperidine	H_2_O	60	60	78–84	[21]
	conventional	[H_2_-DABCO][ClO_4_]_2_	H_2_O	75	15–60	78–95	[22]
	conventional	RuCl_3_·xH_2_O	H_2_O	85	18–120	60–95	[23]
	conventional	Fe_3_O_4_ NPs-cell	H_2_O	—	120	86–96	[24]
	US	CoFe_2_O_4_@SiO_2_/PPA	EtOH	—	3–10	88–96	[25]

**Scheme 3.**
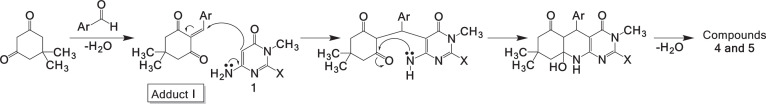
Vilsmeier–Haack formylation of pyrimido[4,5-*b*]quinoline **4** and **5** to yield β-chlorovinyl aldehyde derivatives **6** and **7**.

An important characteristic of MCRs is that certain functionalities present in starting materials can be used in subsequent transformations. Dimedone, provided C6–C7 carbons are in the products, which serve as methylene-active fragments with nucleophilic properties, is suitable for the formation of C–C bonds and the introduction of other functionalities. Thus, derivatives **4** and **5** are suitable precursors for subsequent functionalization and construction of polynuclear heterocycles. This activated carbocycle moiety reacts with VH reagent, enabling the incorporation of a β-chlorovinylaldehyde functionality into pyrimido[4,5-*b*]quinoline derivatives **4** and **5** (scheme 3).

**Scheme 4.**
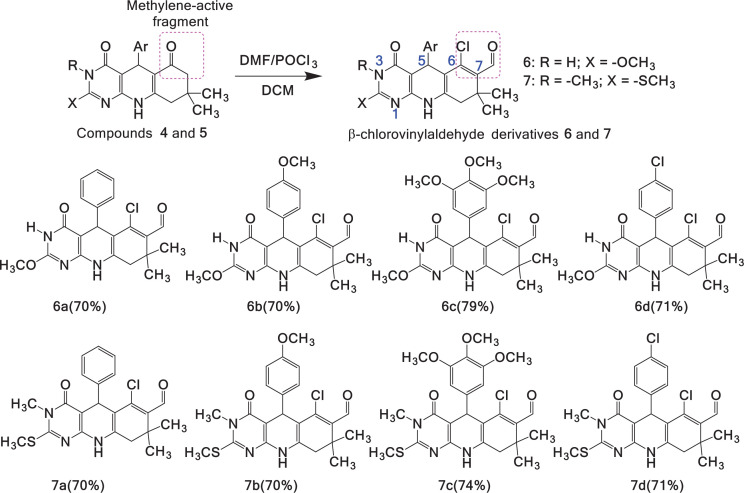
Plausible mechanism for formation of β-chlorovinyl aldehyde derivatives **6** and **7**.

Since the VH reagent was first reported [36], it has positioned itself as a versatile and modern synthetic tool for the introduction of –CHO groups, which undergo transformations, such as halo-formylation, condensation and cyclization [37]. We explored formylation conditions for heterocyclic nuclei [38,39] and hetero-fused systems [40]. The highest yields were achieved using an excess of the VH reagent, specifically on methylene-active fragments with nucleophilic properties. Initially, sonication was used to homogenize the reaction mixture after directly adding pyrimido[4,5-*b*]quinoline derivatives **4** and **5** to the cold VH reagent. Solubility of the substrate was not observed, nor did it progress during the reaction. The solubility of pyrimido[4,5-*b*]quinolindione derivatives is low in alcohols, so it was necessary to solubilize it in a small amount of DCM (0.5 ml), then it was slowly added to the cooled VH reagent and the reaction mixture was sonicated to homogenize. However, significant progress has been observed, which has led to the decision to maintain these conditions and establish a complete sequence of ultrasonically assisted reactions. In this study, regioselective formylation was achieved using a VH reagent with a molar ratio of 2:1 for DMF/POCl_3_. The chloromethyleneiminium salt was prepared by slowly adding DMF dropwise over POCl_3_ while stirring at 0°C and maintaining an argon atmosphere. Subsequently, 1.0 mmol of pyrimido[4,5-*b*]quinoline derivative dissolved in DCM was added over 10 min. The reaction turned red and was USI for 2 h. Under these conditions, chlorovinyl aldehyde derivatives **6** and **7** were obtained in good yield (70–79%). The calculated yields of the pure products are shown in scheme 3, and a possible mechanism for the formation of β-chlorovinyl aldehyde derivatives **6** and **7** is illustrated in scheme 4.

The temperature of the ultrasonic bath used to promote the reactions reported in this study exceeded room temperature. These elevated temperature conditions greatly favoured the one-pot MCR process, completing the reaction within minutes (5–15 min) and isolating products **4** and **5** by filtration and purification via recrystallization from ethanol. In the preparation of VH reagents (formilant agent), it is common to use POCl_3_ as an inorganic acid halide. A chloromethyleneiminium salt, with a known formation mechanism, was prepared using this process [33]. For the formylation of methylene groups under VH conditions, the reaction is typically promoted by heating, and reports have shown that the reactions can be accelerated by microwave and ultrasound radiation [41–44]. In our work, to obtain β-chlorovinyl aldehyde derivatives **6** and **7**, the reaction was not explored under reflux or controlled temperature heating conditions, and ultrasound-assisted conditions allowed the control of HCl release.

**Figure 2.**
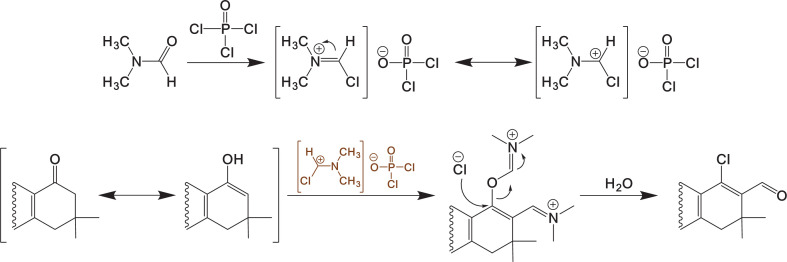
HMBC correlations for compound **7d**.

All β-chlorovinyl aldehyde derivatives **6** and **7** were structurally characterized by ^1^H–^13^C NMR, IR and MS. Structural peculiarities, such as the dihydropyridine ring and the –CHO group, were observed in the ^1^H NMR spectra. In the β-chlorovinyl aldehyde derivatives **6** and **7**, the signals for the protons of the dihydropyridine ring and singlets for Ar–C_5_H and –NH were in the intervals of 4.26–5.31 ppm and 12.23–13.17 ppm, respectively. The signal to the proton –CHO group varies in the interval 9.63–9.68 ppm (electronic supplementary material, figures S1–S8). To corroborate the identification of the different nuclei in compounds **6** and **7**, HMBC spectra of derivative **7d** were obtained (figure 2; electronic supplementary material, figure S9) [45].

**Scheme 5.**
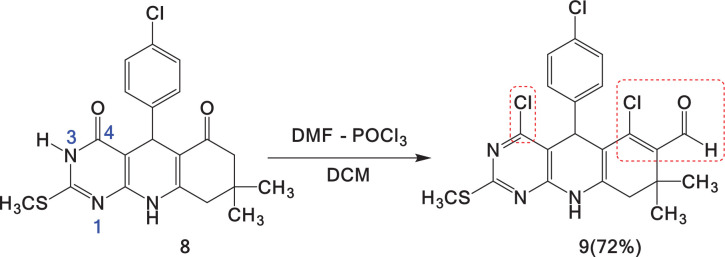
VH formylation and chlorination of pyrimido[4,5-*b*]quinoline **8**.

**Scheme 6.**
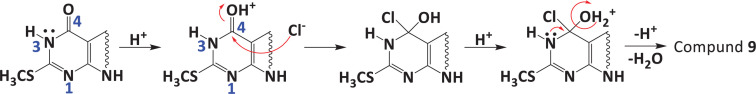
Possible explanation for VH chlorination on pyrimidine ring.

Under these standardized conditions, an experiment was performed using pyrimido[4,5-*b*]quinoline derivative **8** to explore the influence of the VH reagent on the substrates. In addition to formylation, chlorination was observed in C-4 (scheme 5; electronic supplementary material, figure S10; ^1^H and ^13^C NMR spectra for compound **9**) [45]. Unlike the standardized conditions, excess VH reagent was used, which was prepared at a molar ratio of 3:2 for DMF/POCl_3_. Similar conditions were used for the chlorination reactions [46]. The reaction with an excess of the VH reagent leads to β-chlorovinyl aldehyde **9**, which is formed by enolization of the self-catalysed pyrimidine carbonyl group by the action of the released HCl and subsequent nucleophilic substitution by the attack of the chloride ion, water loss and subsequent aromatization of the pyrimidine ring (scheme 6).

**Scheme 7.**
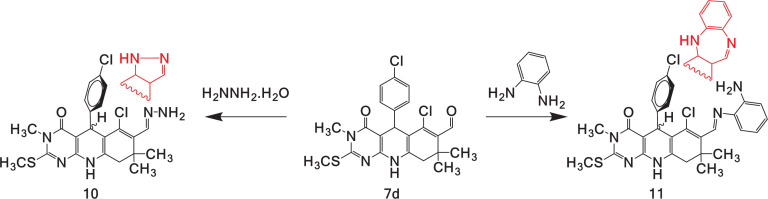
Chlorovinyl imine derivatives.

The ultrasonically assisted reactions described in this study offer a simple, facile and eco-friendly procedure for the efficient synthesis of pyrimido[4,5-*b*]quinoline-4,6-diones and VH formylation, generating stable solids with good efficiency in a few minutes. β‐Chlorovinyl aldehydes are 1,3-bi-electrophilic systems with excellent and useful features for a variety of cyclization and heterocyclization reactions [47–51] with substrates having bi-nucleophilic properties. To experimentally verify this reaction with our β‐chlorovinyl aldehyde derivatives, we initiated a reaction between 6-chloro-5-(4-chlorophenyl)-3,8,8-trimethyl-2-(methylthio)-4-oxo-3,4,5,8,9,10-hexahydropyrimido[4,5-*b*]quinoline-7-carbaldehyde **6d** and hydrazine as the ambident nucleophile. Thus, it is possible to increase the number of heterocyclic rings and structurally diversify polycyclic systems (scheme 7).

The conditions for heterocyclization are simple. The reaction was initiated with amine and β‐chlorovinyl aldehyde **7d** in a 1:1 ratio with stirring, heating at a controlled temperature and reflux using ethylene glycol as the solvent. The progress of the reaction was slow, and the expected product was not obtained; instead, the chlorovinyl imine derivative was isolated in low yield (≤40%). The reaction was induced by microwave radiation for 3 min without using solvents or catalysts and without the formation of the expected compound. Schiff bases were isolated in a good yield (70%). The structures of the synthesized imine derivatives **10** and **11** were established by NMR, IR and MS analyses (electronic supplementary material, figures S11 and S12). To complement the correct assignment of the signals in Schiff bases **10** and **11**, two-dimensional NMR experiments (HSQC and HMBC) were performed on **11** (electronic supplementary material, figures S13 and S14) [45].

Experimental and theoretical studies of pyrimido[4,5-*b*]quinoline-nucleus compounds have suggested kinetic control in heterocyclization [29]. Based on this study, including structural X-ray analysis [35], the conformation-boat of the dihydropyridine ring was demonstrated because of the *sp* ^3^ hybridized C5-atom (stereogenic centre), which addresses the aryl ring to the pseudo-axial position. Orientation due to steric factors may restrict cyclization. Thermodynamic analysis of the reaction was carried out through quantum chemical calculations using Gaussian16 software at the HF/6-311++G(d,p) level to optimize the molecular structure of the compounds [52–54]. These results indicate that the free energy of the reaction from **10** to form the cyclized product is 33.97 times less thermodynamically demanding than the assumed reaction to form the cyclized product (electronic supplementary material, pp. 20–22) [45].

## 4. Conclusion

Ultrasound-assisted synthesis of pyrimido[4,5-*b*]quinoline-7-carbaldehyde derivatives 6 and 7 from pyrimido[4,5-*b*]quinoline-4,6-dione derivatives **4** and **5** is an alternative for the functionalization of heterocyclic systems using the VH reagent. All products were stable at room temperature, and no benzylic oxidation products were obtained during the synthesis and isolation processes. The yields of the heterocyclic compounds did not depend on the type or pattern of substituents. Therefore, the shorter reaction time and lower temperature induced by ultrasound radiation were sufficient to functionalize derivatives **4** and **5**. These results demonstrate a simple methodology for ultrasound-promoted preparation of pyrimido[4,5-*b*]quinoline-based compounds and subsequent formylation using cheap reagents. *Highlights*: (i) simple and efficient synthesis, easy and clean workup, (ii) atom economy and few by-products, (iii) hazardous condition control (POCl_3_-DMF), and (iv) reaction conditions with good performance. Features and benefits of synthetic processes with minimal environmental impact.

## Data Availability

The datasets correspond to the spectra collected for the structural characterization of the compounds reported in this article and are deposited as electronic supplementary material at Figshare [45].

## Dedication

Dedicated to Professor Braulio Insuasty on the occasion of his retirement.
